# Supplementary material for: Seminoma and Embryonal Carcinoma Footprints Identified by Analysis of Integrated Genome-Wide Epigenetic and Expression Profiles of Germ Cell Cancer Cell Lines
Source: PLoS One. 2014 Jun 2;9(6):e98330. doi: 10.1371/journal.pone.0098330 (PMC4041891; doi:10.1371/journal.pone.0098330)
Supplement: File S1 — ZIP file containing DMRforPairs output for significant regions. Please start from the html files. (ZIP) [file pone.0098330.s008.zip › figures/31159.pdf]

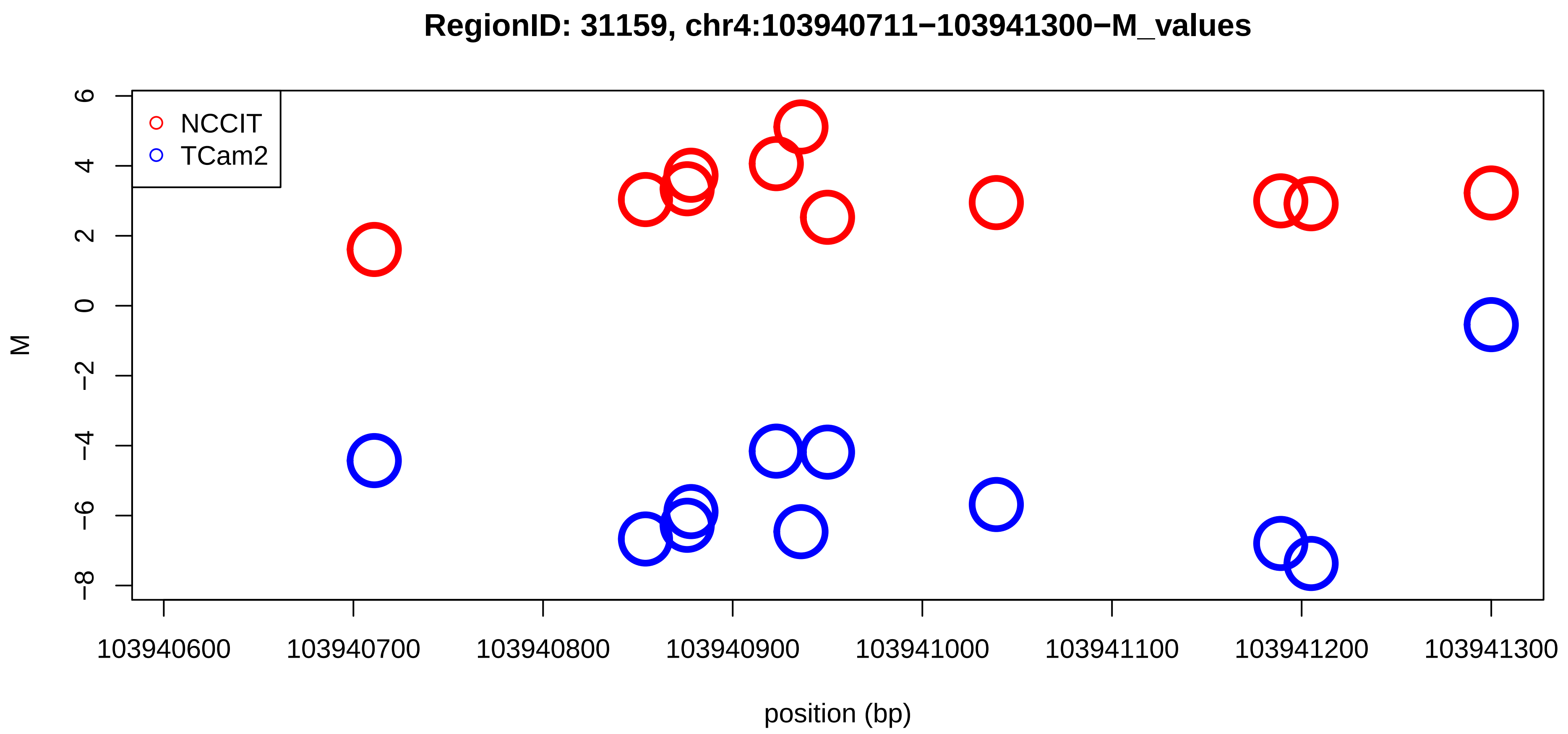
RegionID: 31159, chr4:103940711−103941300−M_values
NCCIT
TCam2
M
6
4
2
0
−2
−4
−6
−8
103940600
103940700
103940800
103940900
103941000
103941100
103941200
103941300
position (bp)

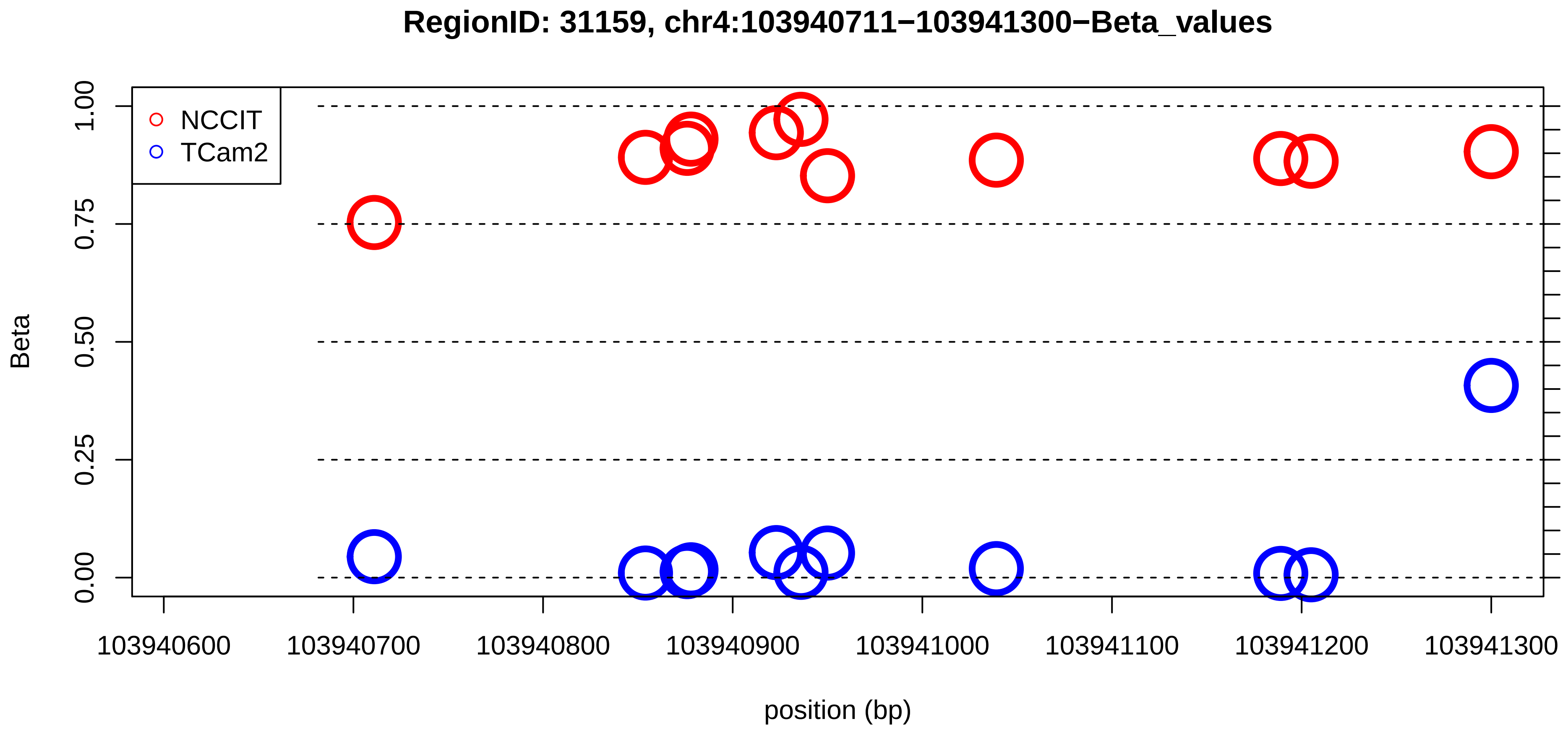
RegionID: 31159, chr4:103940711−103941300−Beta_values
NCCIT
TCam2
Beta
1.00
0.75
0.50
0.25
0.00
103940600
103940700
103940800
103940900
103941000
103941100
103941200
103941300
position (bp)

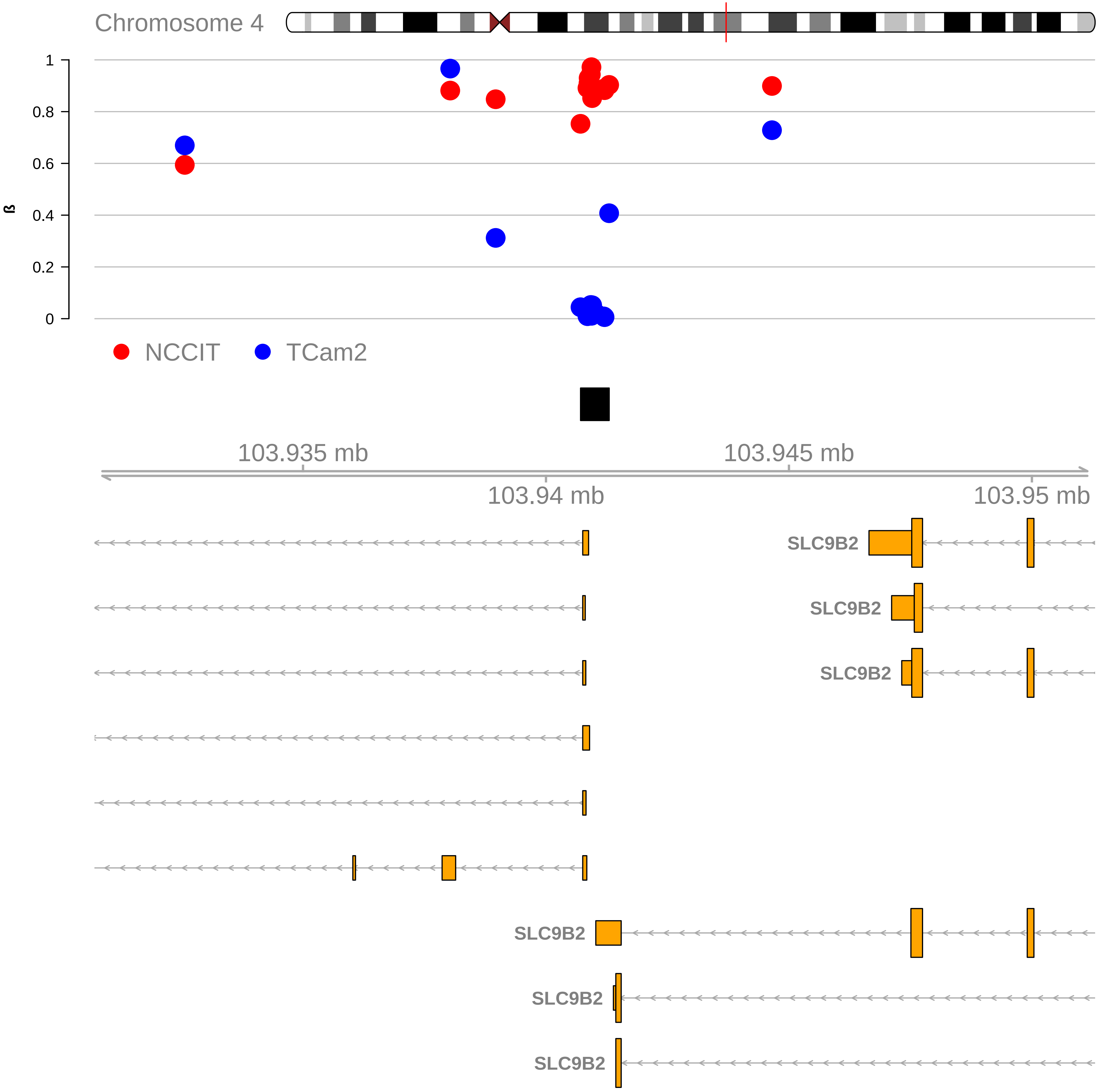

Chromosome 4
β
1
0.8
0.6
0.4
0.2
0
NCCIT
TCam2
103.935 mb
103.945 mb
103.94 mb
103.95 mb
SLC9B2
SLC9B2
SLC9B2
SLC9B2
SLC9B2
SLC9B2
